# The efficacy and toxicity of individualized intensity-modulated radiotherapy based on the tumor extension patterns of nasopharyngeal carcinoma

**DOI:** 10.18632/oncotarget.8004

**Published:** 2016-03-09

**Authors:** Li Lin, Ji-Jin Yao, Guan-Qun Zhou, Rui Guo, Fan Zhang, Yuan Zhang, Lin Xu, Lu-Lu Zhang, Ai-Hua Lin, Jun Ma, Ying Sun

**Affiliations:** ^1^ Department of Radiation Oncology, Sun Yat-Sen University Cancer Center, State Key Laboratory of Oncology in South China, Collaborative Innovation Center for Cancer Medicine, Guangzhou 510060, People's Republic of China; ^2^ Department of Medical Statistics and Epidemiology, School of Public Health, Sun Yat-Sen University, Guangzhou 510080, People's Republic of China

**Keywords:** nasopharyngeal carcinoma, intensity modulated radiotherapy, individualized clinical target volume, clinical outcome, toxicities

## Abstract

**Background:**

To evaluate the efficacy and toxicity of intensity-modulated radiotherapy (IMRT) using individualized clinical target volumes (CTVs) based on the loco-regional extension patterns of nasopharyngeal carcinoma (NPC).

**Methods:**

From December 2009 to February 2012, 220 patients with histologically-proven, non-disseminated NPC were prospectively treated with IMRT according to an individualized delineation protocol. CTV1 encompassed the gross tumor volume, entire nasopharyngeal mucosa and structures within the pharyngobasilar fascia with a margin. CTV2 encompassed bilateral high risk anatomic sites and downstream anatomic sites adjacent to primary tumor, bilateral retropharyngeal regions, levels II, III and Va, and prophylactic irradiation was gave to one or two levels beyond clinical lymph nodes involvement. Clinical outcomes and toxicities were evaluated.

**Results:**

Median follow-up was 50.8 (range, 1.3–68.0) months, four-year local relapse-free, regional relapse-free, distant metastasis-free, disease-free and overall survival rates were 94.7%, 97.0%, 91.7%, 87.2% and 91.9%, respectively. Acute severe (≥ grade 3) mucositis, dermatitis and xerostomia were observed in 27.6%, 3.6% and zero patients, respectively. At 1 year, xerostomia was mild, with frequencies of Grade 0, 1, 2 and 3 xerostomia of 27.9%, 63.3%, 8.3% and 0.5%, respectively.

**Conclusions:**

IMRT using individualized CTVs provided high rates of local and regional control and a favorable toxicity profile in NPC. Individualized CTV delineation strategy is a promising one that may effectively avoid unnecessary or missed irradiation, and deserve optimization to define more precise individualized CTVs.

## INTRODUCTION

Nasopharyngeal carcinoma is most endemic in South-Eastern Asians, with an age-standardized incidence in male of 20–50/100000 in southern china [1]. Radiation therapy is the mainstay treatment modality for non-metastatic disease. For decades, NPC radiation therapy utilizes two-dimensional conventional treatment (2D-CRT). Disease control has been acceptable [2–4]; however, high-dose irradiation has been associated with high probability of toxicities. Currently, intensity modulated radiotherapy (IMRT) is generally accepted as a more advanced radiation technique to improve the therapeutic ratio and encouraging outcomes have been achieved over the past decade [5–8].

The delineation of ideal target volumes is absolutely one of the most critical procedures in IMRT. However, optimal clinical target volume (CTV) for NPC is far away from determined. In the current practise of most institutions, anatomic sites surrounding the nasopharynx were empirically delineated bilaterally as CTV, regardless of the tumor extension patterns. Even though the CTV vary significantly among institutions, the local and regional control were comparably satisfactory, and outside-field loco-regional failures were fairly rare [9–14]. We speculate that current CTVs are large enough to encompass microscopic spread; but unnecessary irradiation and side effects may be existed [15–17]. With the improved survival in modern diagnostic and therapeutic modality of NPC, adequate quality of life after treatment is essential for patients and their family. Consequently, optimal target definition in 3D planning of IMRT is imperative to transform the technical advantages of IMRT to improved efficacy and toxicity profile.

In addition, current CTV delineation largely originated from experience in 2D-CRT rather than the loco-regional extension patterns of this malignancy. Our previous studies have investigated the patterns of local extension and cervical lymph node (LN) metastasis [18, 19], which was fundamental in understanding the biological characteristics of NPC and could help to define individualized CTV avoiding unnecessary or missed irradiation.

Here, we generated an individualized CTV delineation protocol based on the loco-regional extension patterns of NPC. The aim of this work was to report the efficacy and toxicity of a cohort of patients perceptively treated with individualized IMRT and to explore the feasibility of individualized CTV delineation in NPC.

## RESULTS

### Dose-volume analysis for targets and organs at risk

Table 1 shows the dose–volume statistics for targets and organs at risk (OARs). The mean dose to planning target volume of nasopharynx (PTVnx) was 72.62 (range, 70.77–74.52) Gray (Gy). On average, target volumes had excellent dosimetric distributions. The prescribed dose encompassed 98.84% of PTVnx, 99.43% of planning target volume 1 (PTV1) and 98.54% of planning target volume 2 (PTV2); only 5.33% of PTVnx received ≥ 110% of the prescribed dose. The dose received by 1% of the volume of the planning organ at risk volume (PRV) of the brain stem and spinal cord was 58.64 Gy and 39.87 Gy, respectively. The doses delivered were within the tolerance limits of most OARs, except bilateral parotid glands.

**Table 1:** Dose-volume data for the targets and critical organs at risk (OARs)

	Structure	Parameters (unit)	Constraints	Result Mean (range)	Dmean (Gy) Mean (range)	Volume (cc)^#^ Mean (range)
**Targets**	PTVnx	V95% (%)*	≥ 99	99.85 (96.9–100.00)	72.62 (70.77–74.52)	44.71 (0.89–246.60)
		V100% (%)*	≥ 95	98.84 (94.40–100.00)		
		V110% (%)*	≤ 20	5.33 (0–44.6)		
	PTV1	V95% (%)	≥ 99%	99.92 (99.00–100.00)	68.76 (65.45–71.33)	124.07 (21.21–448.86)
		V100% (%)	≥ 95%	99.43 (96.90–100.00)		
	PTV2	V95% (%)	≥ 99%	99.70 (96.90–100.00)	62.44 (58.75–66.37)	531.81 (248.10–1163.80)
		V100% (%)	≥ 95%	98.54 (91.91–99.94)		
**OARs**	Brainstem	Dmax (Gy)†	≤ 54	54.10 (40.95–69.40)		
	Brainstem PRV	D1% (Gy)††	≤ 60	58.64 (47.94–74.40)		
	Spinal cord	Dmax (Gy)	≤ 45	37.92 (33.38–48.95)		
	Spinal cord PRV	D1% (Gy)	≤ 50	39.87 (35.00–51.18)		
	Left optic nerve	Dmax (Gy)	≤ 54	41.37 (4.33–73.72)		
	Right optic nerve	Dmax (Gy)	≤ 54	40.46 (4.54–70.55)		
	Chiasm	Dmax (Gy)	≤ 54	50.08 (10.59–74.48)		
	Left temporal lobe	Dmax (Gy)	≤ 60	60.15 (41.97–74.95)		
	Right temporal lobe	Dmax (Gy)	≤ 60	59.93 (42.10–75.47)		
	Left parotid	Dmean (Gy)‡	≤ 26	39.18 (28.94–63.20)		
		V30Gy (%)¶	≤ 50	60.03 (24.60–100.00)		
	Right parotid	Dmean (Gy)	≤ 26	38.90 (30.14–61.22)		
		V30Gy (%)	≤ 50	58.54 (29.20–100)		

* Percentage volume receiving ≥ 95%, 100% or 110% of the prescribed dose;

† Maximum dose;

†† Dose received by 1% of the volume concerned;

‡ Mean dose;

¶ Percentage volume receiving ≥ 30 Gy;

# Volumes of GTVnx, CTV1 and CTV2.

### Treatment outcomes

**Figure 1:**
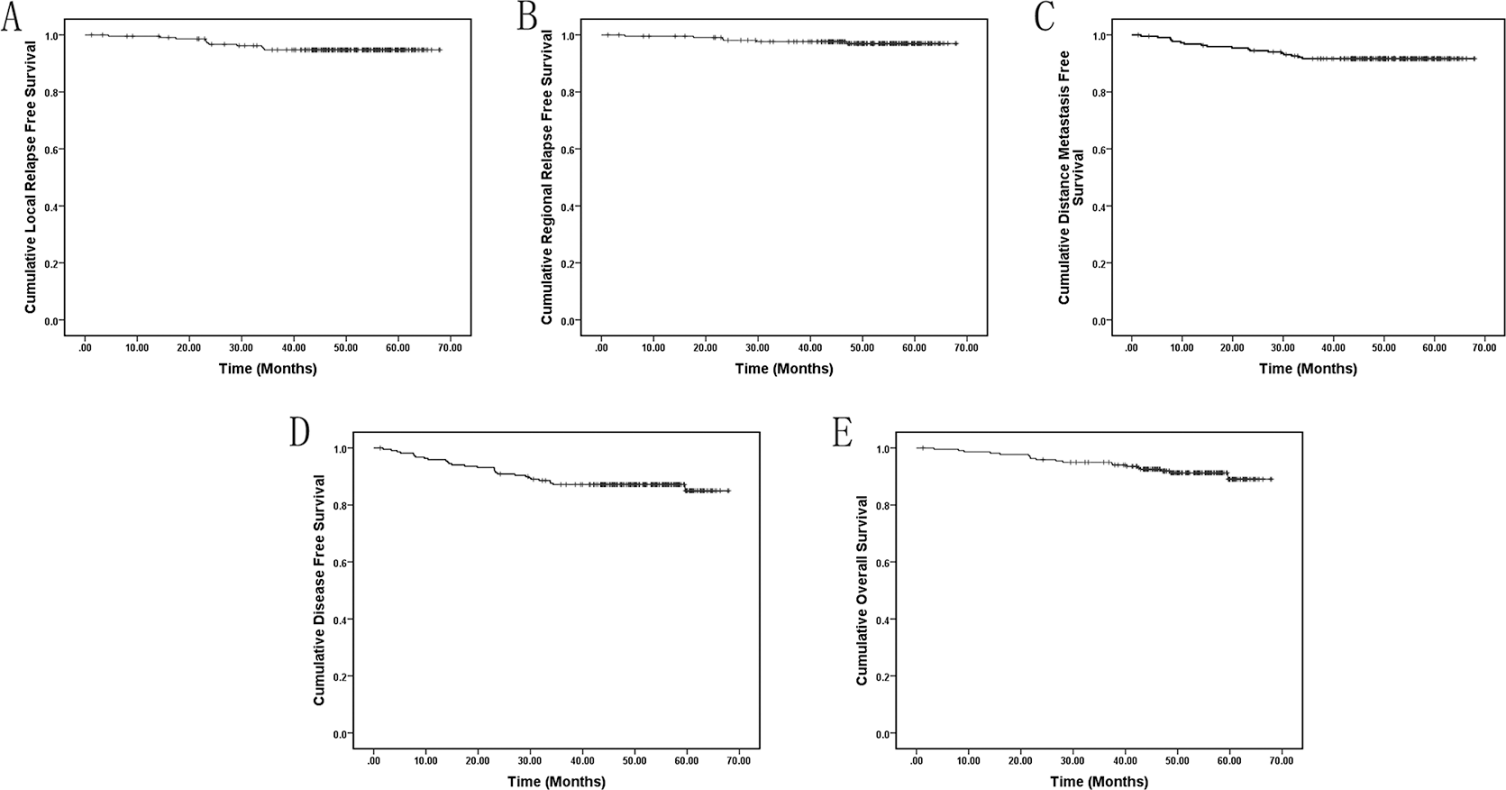
Kaplan-Meier local relapse free (A), regional relapse-free (B), distant metastasis-free (C), disease-free (D), and overall (E) survival curves

All patients completed the scheduled radiotherapy. The median follow-up was 50.8 (range, 1.3–68.0) months, 58.7% of patients were followed up for more than 4 years. Of the patients, 11 developed local relapse; 6, regional relapse; 18, distant metastasis; and 19 died. Four-year estimated local relapse free survival (LRFS), regional relapse free survival (RRFS), distant metastasis free survival (DMFS), disease free survival (DFS) and overall survival (OS) rates were 94.7% (95% Confidence Interval, CI: 91.8%–97.6%), 97.0% (95%CI: 94.7%–99.4%), 91.7% (95%CI: 87.9%–95.4%), 87.2% (95%CI: 82.7%–91.7%) and 91.9% (95%CI: 88.2%–9.6%), respectively (Figure 1). Thirteen deaths were due to distant metastasis; 5, progression of loco-regional disease after recurrence; and 1, an unrelated accident.

Median time to loco-regional recurrence was 23.7 (range, 4.5–34.3) months. Ten of 11 cases with local recurrence occurred within the 95% isodose lines of PTVnx and were considered in-field failures; the other case recurred at the initial primary site with extension out of PTVnx and was considered marginal failure. No recurrence was observed outside the margin of CTV2. MRI images obtained before treatment and at the time of recurrence of primary tumor of 11 patients with local recurrence are show in Supplementary Figure S1A–S1E. Most local relapses (9/11) occurred in patients with locally advanced disease.

Of the 6 regional recurrences, 5 occurred in level IIb where metastatic nodes were formerly present; the other, in level Ia and Ib that haven't been irradiated before at 23.3 months, and was considered out-of-field failure. Five of the 6 regional recurrences occurred with local recurrence. Four–year RRFS rates of N0 and LN-positive patients were 100% and 96.5%, respectively.

### Prognostic factors

The value of potential prognostic factors including age, gender, T classification, N classification, WHO histological grade and chemotherapy were evaluated using Cox proportional hazards models with backward elimination. The T classification was found to be an independent predictor for OS (*P* = 0.008; HR = 2.29; 95% CI = 1.24–4.214), whereas all the other prognostic factors were found to be insignificant (Table 2).

**Table 2:** Multivariate analyses of prognostic factors

Variable	HR (95% CI), *P*-value				
	LRFS	RRFS	DMFS	DFS	OS
Gender, male *vs.* female	1.28 (0.22–7.56)	1.51 (0.43–5.34)	0.61 (0.18–2.12)	0.93 (0.39–2.20)	0.56 (0.16–1.94)
	*P* = .78	*P* = .53	*P* = .44	*P* = .86	*P* = .36
Age, ≥ 50 *vs.* < 50 years	1.11 (0.20–6.28)	0.53 (0.11–2.43)	1.42 (0.55–3.68)	1.35 (0.63–2.87)	1.77 (0.71–4.42)
	*P* = .90	*P* = .41	*P* = .47	*P* = .44	*P* = .22
T classification, T4 *vs.* T3 *vs.* T2 *vs.* T1	0.88 (0.39–1.99)	1.06 (0.57–1.96)	1.44 (0.86–2.43)	1.32 (0.89–1.97)	2.29 (1.24–4.21)
	*P* = .75	*P* = .85	*P* = .17	*P* = .17	*P* = .008
N classification, N3 *vs.* N2 *vs.* N1 *vs.* N0	1.60 (0.84–3.04)	1.46 (0.87–2.44)	1.24 (0.80–1.92)	1.37 (0.99–1.90)	1.37 (0.92–2.03)
	*P* = .15	*P* = .15	*P* = .34	*P* = .056	*P* = .12
WHO histological grade, Type III *vs.* Type I and II	—	—	1.53 (0.34–6.89)	0.83 (0.20–3.54)	0.48 (0.06–3.78)
	—	—	*P* = .58	*P* = .81	*P* = .49
Chemotherapy, with *vs.* without	0.50 (0.04–5.67)	0.90 (0.10–8.50)	1.98 (0.24–16.22)	0.86 (0.24–3.16)	0.56 (0.11–2.63)
	*P* = .58	*P* = .92	*P* = .52	*P* = .83	*P* = .46

Abbreviations: HR, hazards ratio; 95% CI, 95% confidence interval; RT, radiotherapy; CRT, chemoradiotherapy; LRFS, local relapse free survival; RRFS, regional relapse free survival; DMFS, distance metastasis free survival; DFS, disease free survival; OS, overall survival.

### Acute and late radiation toxicities

Acute toxicities were assessed during treatment in 220 patients and late toxicities in 218 patients with ≥ 6 months follow-up (Table 3). Acute toxicities of radiotherapy ± chemotherapy were well tolerated. Acute severe (≥ Grade 3) mucositis, dermatitis and xerostomia were observed in 27.6%, 3.6% and zero patients, respectively. Xerostomia at 1 year was mild, with frequencies of Grade 0, 1, 2 and 3 xerostomia of 27.9%, 63.3%, 8.3% and 0.5%, respectively. Fourteen patients (6.4%) developed temporal lobe injury diagnosed by MRI after IMRT.

**Table 3:** Frequencies of the most common acute and late radiation toxicities by type and grade

Treatment toxicities	Total	Grade 1	Grade 2	Grade 3	Grade 4
**Acute toxicities, *n (%)***					
Mucositis	219 (99.0)	53 (24.0)	105 (47.4)	61 (27.6)	0
Dermatitis	215 (97.0)	146 (65.8)	61 (27.6)	8 (3.6)	0
Xerostomia	205 (92.8)	143 (64.8)	62 (28.0)	0	0
**Late toxicities, *n (%)***					
Xerostomia*	157 (72.1)	138 (63.3)	18 (8.3)	1 (0.5)	0
Hearing loss	71 (32.6)	67 (30.7)	3 (1.4)	1 (0.5)	0
Neck fibrosis	67 (30.7)	67 (30.7)	0	0	0
Radiation encephalopathy	14 (6.4)	13 (5.9)	1 (0.5)	0	0

* At 12 months after radiotherapy, 217 patients were evaluable.

## DISCUSSION

This report summarizes the efficacy and toxicity of IMRT using an individualized CTV delineation protocol based on the patterns of tumor extension and biological characteristics of NPC. Up to now, the optimal CTV delineation scheme remains to be identified, and our work was the first attempt to explore the feasibility of individualized CTV delineation during IMRT planning. Using individualized CTV, we achieved a 4-year LRFS, RRFS, DMFS, DFS and OS of 94.7%, 97.0%, 91.7%, 87.2% and 91.9%, respectively, comparable to the historical data [9–14]. Excellent outcomes supported the feasibility of individualized CTV delineation.

### Feasibility of individualized CTV delineation for local disease

It is difficult to gain pathological confirmation of tumor extension in NPC as it is unresectable. Thus, imaging concerned tumor extension patterns are critical for CTV delineation. According to the cumulative incidences of tumor invasion, Liang et al. and Li et al. classified the anatomic sites surrounding the nasopharynx into three risk grades [18, 20]. The high risk anatomic sites are all adjacent to the nasopharynx and have a cumulative involvement probability > 30%. Thus, it is reasonable to speculate that these high risk anatomic sites are indeed at high risk of tumor invasion; and should be included in CTV2 in all patients. As the risk of tumor invasion gradually reduces with the distance from the nasopharynx, it seems reasonable to subdivide the CTV into CTV1 and CTV2 with different dose levels to different risk regions. In many treatment centers, only one CTV for the primary tumor is delineated, and the region is similar to that of our CTV2; however, the dose prescribed is the same as that to our CTV1 [9–11, 14]. The comparable local control rates indicate it is safe to narrow the area irradiated at 60 Gy and prescribe a lower dose to CTV2.

Medium and low risk anatomic sites are separated from the nasopharynx by other anatomic sites and are rarely invaded without involvement of adjacent high or medium risk anatomic sites [18, 20]. In most studies, medium or low risk anatomic sites including the bilateral foramen ovale, pterygopalatine fossa, sphenoid sinus, and posterior part of the maxillary sinus are regularly included in CTV. This strategy seems unreasonable, especially in T1 or T2 disease, due to the risk of unnecessary irradiation. In current practice, prophylactic radiation is prescribed along the tumor infiltration routes to include downstream anatomic sites adjacent to the tumor, in order to achieve individualized treatment. Accordingly, the aforementioned medium or low risk anatomic sites would be omitted in patients with tumors restricted to the pharyngobasilar fascia. In current series, ten patients experience local recurrence within the delineated GTV, and 1 patient have marginal local recurrence with a component of recurrent foci in the GTV. Isolated recurrence at the edge of the delineated CTV2 was not seen. Individualized CTV for primary disease described above seems to be feasible for radiation therapy for NPC using IMRT.

### Feasibility of individualized CTV delineation for regional disease

Due to the high probability of cervical lymph node metastasis [21], irradiation of the entire neck down to the supraclavicular fossae (SCF) is commonly-accepted practice, even in N0 disease [22]. Recent studies reported that metastasis to the cervical LNs follows orderly pattern, with rarely skipping LN levels [19, 20, 23]. Consequently, it is reasonable to question the necessity of elective irradiation to the lower neck (such as levels IV and Vb) and SCF nodes in patients with N0 disease. Tang et al. retrospectively compared 138 patients had N0 disease with (101 patients) or without (37 patients) lower neck irradiation [19]. None of the patients in either group experienced regional failure, and the risks of distant metastasis did not differ statistically. Furthermore, in a study reported by Gao et al., 410 NPC patients with N0 disease were treated with conventional radiotherapy, but only lymph nodes in the upper neck nodes were electively irradiated [24]. At 5-year follow-up, only one case (0.2%) of regional recurrence in the lower neck was observed. In current series, 30 patients had N0 disease and were irradiated only to the caudal border of the cricoid bone, no patient experienced regional failure. These results suggested that in patients with N0 disease, spearing the lower neck may be acceptable with a minimal risk of regional recurrence. However, further investigations are needed to confirm the findings before the limited neck field becomes the standard of CTV delineation in IMRT for NPC patients with N0 disease.

Currently, most studies advocate treatment of whole neck including level IV, Vb, and SCF in patients with LN-positive patients, regardless of where the nodes were emerged [3, 12–14], Owing to the low risk of “skip metastasis” in NPC, we prophylactically irradiated one or two levels beyond the clinical extent of LN involvement. The 3-year RRFS rate for the LN-positive patients was 97.2%, and none of the regional recurrences occurred in the spared region, although recurrence at level Ia and Ib were observed in one patient. As local recurrence coexisted, this case cannot exactly be classified as regional recurrence as previous radiotherapy may have modified lymphatic drainage. Most recently, Chen et al. reviewed 154 patients with only RLN metastasis, of whom 54 received partial neck irradiation to levels II, III and Va and 100 received whole neck irradiation [25]. The 5-year RRFS and DFS rates for the partial neck irradiation and whole neck irradiation groups were 98.1% vs. 98.0% (*P* = .882) and 87.0% vs. 77.0% (*P* = .117). These results indicate that prospective studies aiming at determining the individualized prophylactic neck irradiation area according to the location of lymph nodes is quite imperative.

Using individualized CTVs, we achieved relatively good target coverage and normal tissue sparing, except for bilateral parotid glands. Although doses irradiated to both parotid glands exceeded the dose limit suggest by the RTOG [26], sever acute and late xerostomia was rare. The explanation may be that we contoured and evaluated the whole parotid gland, including the part overlapped the CTV. Other toxicities of radiotherapy ± chemotherapy were also acceptable, even with extensive use of chemotherapy (86.4%). These may due to good normal tissue sparing and appropriate supportive care.

Despite the favorable local and regional control, there is an important issue need to be addressed. Most anatomic sites surrounding the nasopharynx have considerable volumes, and tumor invasion of these anatomic sites can vary from microscopic foci to gross infiltration. It remains unclear if it is necessary to prophylactically irradiate the adjacent downstream sites when a small proportion of an anatomic site is invaded. To figure out this issue, we now launched a program, attempting to define the invasion risk for each pixel of the anatomic sites surrounding the nasopharynx, thus more precise individualized CTVs could be defined.

To summarize, our individualized CTV delineation protocol resulted in favorable clinical outcomes and is a promising strategy that may effectively avoid unnecessary or missed irradiation. Further optimization is warranted to provide more precise individualized CTVs and maximize the tumor killing and normal tissue protecting effect.

## MATERIALS AND METHODS

### Patients

From December 2009 to February 2012, 220 patients with newly histologically-proven, non-disseminated NPC were prospectively treated with IMRT according to individualized CTV delineation protocol. Eligible patients were aged 18–70 years with histologically proven stage I to stage IVB nasopharyngeal carcinoma (according to the 7th edition of International Union Against Cancer (UICC)/American Joint Committee on Cancer (AJCC) staging system). All patients had Karnofsky scores of at least 70, and adequate bone marrow, liver, and renal function. Our exclusion criteria included previous chemotherapy, radiotherapy, or definitive surgery of the primary tumor or lymph node. We also excluded patients with previous malignancy, with present other active cancer, who were pregnant or lactating, who had unstable cardiac disease needing treatment, or who had hearing loss due to sensorineural deafness. The study was approved by the institutional review board (IRB) and all patients provided written informed consent. Clinicopathological characteristics are summarized in Table 4.

**Table 4:** Clinicopathological characteristics of the entire series of 220 patients

Characteristics	*n* (%)
**Patient factors**	
Age (years)	
≥ 50	68 (30.9)
< 50	152 (69.1)
Gender	
Male	167 (75.9)
Female	53 (24.1)
**Tumor factors**	
Histology	
WHO type I	2 (0.9)
WHO type II	15 (6.8)
WHO type III	203 (92.3)
Clinical stage	
I	10 (4.5)
II	46 (20.9)
III	106 (48.2)
IV	58 (26.4)
T category	
T1	39 (17.7)
T2	33 (15.0)
T3	103 (46.8)
T4	45 (20.5)
N category	
N0	30 (13.6)
N1	132 (60.0)
N2	40 (18.2)
N3	18 (8.2)
**Treatment factors**	
chemotherapy	
Yes	190 (86.4)
No	30 (13.6)

Abbreviations: WHO = World Health Organization;

IMRT = Intensity-modulated radiotherapy.

All patients underwent pre-treatment evaluations including complete history, physical and endoscopic examination, hematology and biochemistry profiles, plasma Epstein-barr virus (EBV) DNA concentrations, magnetic resonance imaging (MRI) of nasopharynx and neck, electrocardiogram, chest radiography, abdominal ultrasonography, emission computed tomography (ECT) and dental evaluation. The 7th edition of International Union Against Cancer (UICC)/American Joint Committee on Cancer (AJCC) staging system was used for disease staging [27].

### IMRT techniques

Patient setup and treatment planning CT scan were performed as formerly described [28]. Target volumes were delineated slice-by-slice on treatment planning CT scans according to an individualized delineation protocol, in accordance with International Commission on Radiation Units and Measurements reports 50 and 62 [29, 30].

Gross tumor volume (GTV) was determined from imaging, physical examination and endoscopy. The primary tumor along with enlarged retropharyngeal lymph nodes (RLNs) was defined as GTVnx, and the involved cervical LNs volume as GTVnd. Organs at risk (OARs) were contoured consistent with Sun's recommendation [31].

**Figure 2:**
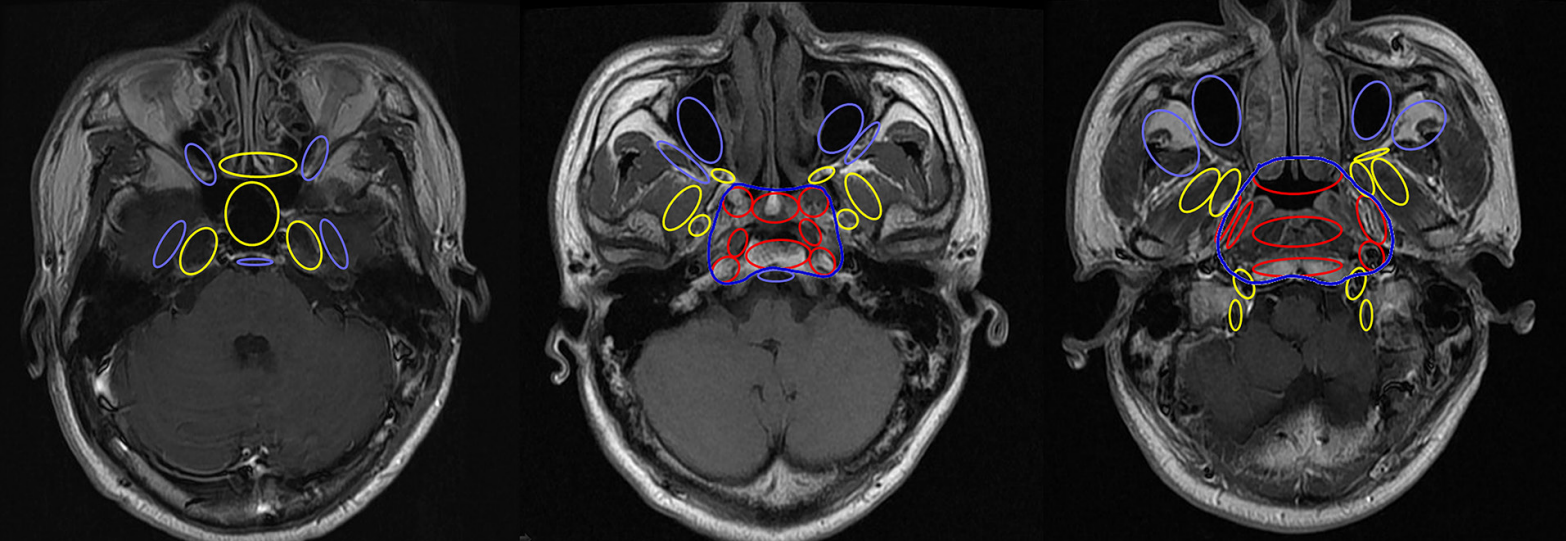
Relationship between the CTV2 and risk of tumor extension Anatomic sites shown in red are at high risk of tumor invasion; yellow, medium risk; and light blue, low risk. The dark blue line represents the smallest CTV2 area for primary tumor.

**Figure 3:**
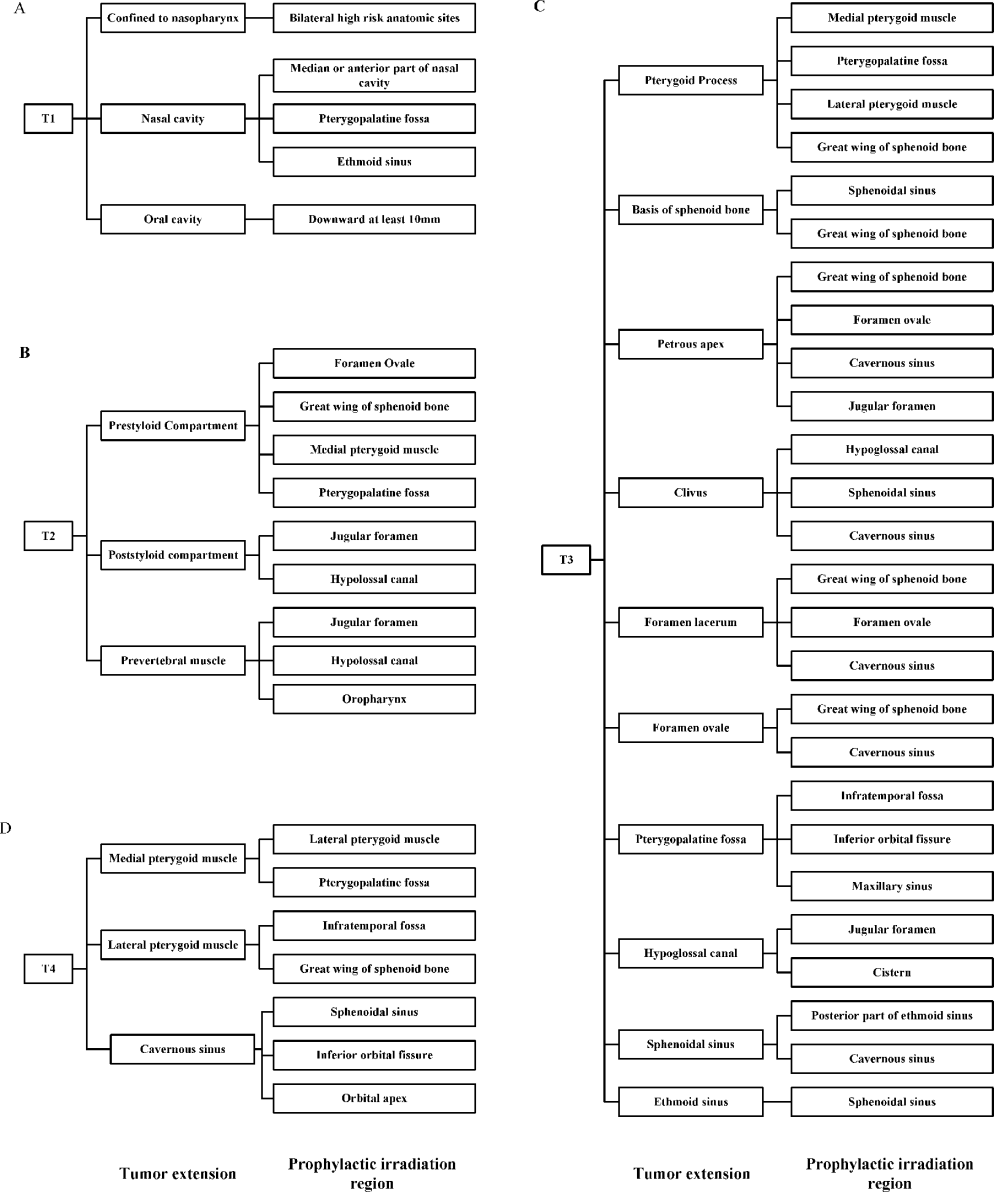
Downstream anatomic sites to be prophylactically irradiated based on T classification and routes of tumor extension T1 (**A**), T2 (**B**), T3 (**C**), T4 (**D**).

Clinical target volume (CTV) was delineated individually based on GTV, patterns of loco-regional extension and biological characteristics of NPC. The CTV for GTVnx included CTV1 for high risk regions and CTV2 for low risk regions of microscopic infiltration. CTV1 was defined as GTVnx plus a 5–10 mm margin, including the entire nasopharyngeal mucosa and structures within the pharyngobasilar fascia. For CTV2, firstly, bilateral anatomic sites at high risk, including parapharyngeal space, posterior part of nasal cavity, pterygoid process, prevertebral muscle, clivus, petrous apex, foramen lacerum and basis of sphenoid bone, were included in each patient (Figure 2). Then, downstream anatomic sites adjacent to involved sites along routes of tumor infiltration were prophylactically irradiated (Figure 3). Finally, the margin of both CTVs could be reduced to 2–3 mm at the sites of brain stem, spinal cord and temporal lobes and margins could be limited to exclude bone or air spaces not at risk of subclinical disease.

Prophylactic neck irradiation areas were also defined as CTV2; individualized irradiation was achieved by selective irradiation according to the patterns of LN metastasis and modes of anatomic lymphatic drainage [19, 20, 22, 32]. Cervical LN levels were defined according to International Consensus Guidelines for LN levels [22, 32]. Firstly, the bilateral retropharyngeal regions, levels II, III, and Va were included in CTV2 in each patient. Then, prophylactic irradiation was given to one or two levels beyond the clinical extent of LN involvement in LN positive patients. That is, ipsilateral levels IV and Vb were included in CTV2 in LN-positive patients and ipsilateral SCF was irradiated in patients with lymphadenopathy beyond the caudal end of the hyoid bone. Finally, level Ib was only electively irradiated if: (1) level Ib LNs were involved, (2) there was extensive nodal disease on the ipsilateral IIa/IIb region or extracapsular extension of level IIa LNs, or (3) the hard palate or ipsilateral nasal cavity was grossly involved. The outermost boundary of the CTV2 should be at least 5 mm from GTVnd.

Planning target volumes (PTVnx, PTVnd, PTV1 and PTV2) were constructed automatically by expanding the corresponding target volumes (GTVnx, GTVnd, CTV1 and CTV2) in three dimensions by 3 mm, allowing for setup variability. All the PTVs should not go outside of the skin surface. A 3 mm margin was added to the brainstem and spinal cord to form the planning organ at risk volume (PRV).

The prescribed doses were 68–70 Gy at 2.12–2.27 Gy/fraction for PTVnx, 64–70 Gy at 2.00–2.13 Gy/fraction for PTVnd, 60 Gy at 1.82–2.00 Gy/fraction for PTV1, and 54–56 Gy at 1.70–1.80 Gy/fraction for PTV2. The plan could be accepted if the target coverage met the criterion and dose receive by OARs should be as low as possible to met the restrictions (Table 2) [26]. All targets were treated simultaneously using the simultaneous integrated boost (SIB) technique. Treatment was delivered with a computer-controlled auto sequence multi-leaf collimator (MLC) on a linear accelerator. All treatments were delivered once daily, 5 days per week.

### Chemotherapy

During the study, institutional guidelines recommended IMRT only for stage I and concurrent chemoradiotherapy ± neoadjuvant/adjuvant chemotherapy for stage II to IVB. Overall, 30 (13.6%) patients were treated with IMRT alone, and 190 (86.4%) received chemotherapy. Of the 164 patients with stage III/IV disease, 155 (94.5%) received chemotherapy Reasons for deviation included advanced age, organ dysfunction or allergic reactions. Neoadjuvant or adjuvant chemotherapy consisted of cisplatin with 5-fluorouracil and/or docetaxel every three weeks for two or three cycles. Concurrent chemotherapy consisted of cisplatin weekly or on days 1, 22 and 43 of radiotherapy.

### Follow-up and statistical analysis

All patients were evaluated at least per week during treatment and followed-up at least every three months in the first 2 years, every 6 months in the subsequent 3 years, and every year thereafter. Routine follow-up care included fiberoptic endoscopic examination, MRI of nasopharynx and neck, chest radiography, abdominal sonography, ECT and plasma EBV DNA concentrations.

Local relapse-free survival (LRFS), regional relapse-free survival (RRFS), distant metastasis-free survival (DMFS), disease-free survival (DFS) and overall survival (OS) rates were estimated by the Kaplan-Meier method. Durations were calculated from start of treatment. Radiotherapy-related toxicities were graded using the Acute and the Late Radiation Morbidity Scoring Criteria of the Radiation Therapy Oncology Group [33]. Multivariate analysis was performed using a Cox proportional hazards model. Analyses were performed using Statistical Package for the Social Sciences version 17.0 (SPSS; Chicago, IL, USA). Two-tailed *P*-values ≤ 0.05 were considered significant.

## SUPPLEMENTARY MATERIALS FIGURE


