# Supplementary material for: The efficacy and toxicity of individualized intensity-modulated radiotherapy based on the tumor extension patterns of nasopharyngeal carcinoma
Source: Oncotarget. 2016 Mar 9;7(15):20680–90. doi: 10.18632/oncotarget.8004 (PMC4991484; doi:10.18632/oncotarget.8004)
Supplement: Supplementary file 1 [file oncotarget-07-20680-s001.pdf]

The efficacy and toxicity of individualized intensity-modulated radiotherapy based on the tumor extension patterns of nasopharyngeal carcinoma

Supplementary Materials

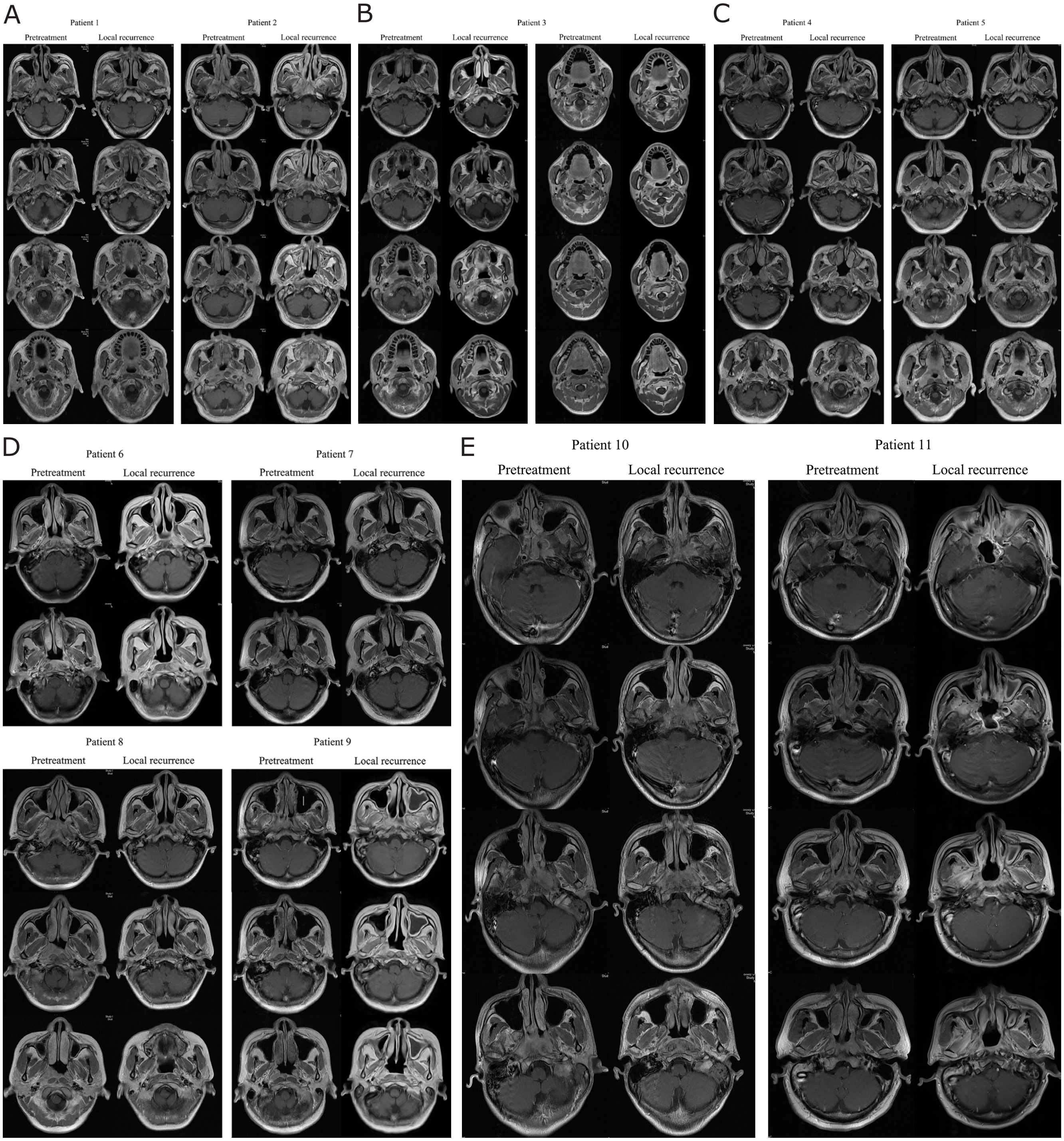

Supplementary Figure S1: MRI images obtained before treatment and at the time of recurrence of primary tumor of 11 patients with local recurrence. (**A**) patient 1 and 2; (**B**) patient 3; (**C**) patient 4 and 5; (**D**) patient 6, 7, 8 and 9; (**E**) patient 10 and 11. Patients 1 to 10 were identified as in-field failures, and patient 11 was identified as marginal failure.
